# Supplementary material for: Ex Vivo Culture Models to Indicate Therapy Response in Head and Neck Squamous Cell Carcinoma
Source: Cells. 2020 Nov 23;9(11):2527. doi: 10.3390/cells9112527 (PMC7700693; doi:10.3390/cells9112527)
Supplement: Supplementary file 1 [file cells-09-02527-s001.zip › Table S1.docx]

**Table S1.** Overview of included studies describing ex-vivo culture models of HNSCC for chemotherapy and radiotherapy sensitivity testing.

| Authors,  year | | Culture technique | | Patients  (N) | | Culture duration  (days) | | Culture  success  (%) | | Ex-vivo treatment | | Response read-out method | Preservation of tissue parameters in culture | | Main results of treatment | | In-patient treatment | | Correlation ex-vivo *vs* in-patient | |
| --- | --- | --- | --- | --- | --- | --- | --- | --- | --- | --- | --- | --- | --- | --- | --- | --- | --- | --- | --- | --- |
| Leong,  2014  [51] | | Multicellular Spheroid | | 3 | | 4-9 | | - | | Cisplatin,  5-FU,  Etoposide,  RT | | FACS | - | | Spheroids were more resistant to all treatments than monolayers.  Cells with a high ALD expression were resistant to cytotoxic agents. | | - | | - | |
| Hagemann,  2017  [22] | | Multicellular Spheroid | | - | | 15 | | - | | Cisplatin,  5-FU,  RT | | Spheroid area and diameter,  ELISA | In preliminary data, primary cells did not form reproducible spheroids in hanging-drop but did in ultra-low attachment plates. | | Cisplatin, 5-FU, and RT significantly decrease spheroid growth  Combination of RT and cisplatin reduced number of viable cells but not spheroid size. | | - | | - | |
| Lim,  2011  [24] | | CSC-enriched spheroids | | 47 | | 14 | | 6% | | Cisplatin,  5-FU,  Paclitaxel,  Docetaxel | | MTT | - | | Undifferentiated spheroid cells were significantly more resistant to all chemotherapeutic agents than differentiated spheroid cells. | | - | | - | |
| Lim,  2012  [25] | | CSC-enriched spheroids | | 31 | | 15-17 | | - | | Cisplatin,  5-FU,  Paclitaxel,  Docetaxel | | MTT | - | | Cell survival:  Control 100%  Cisplatin: 70%  5-FU: 60%  Paclitaxel: 55%  Docetaxel: 60% | | - | |  | |
| Oh,  2013  [104] | | CSC-enriched spheroids | | - | | >14 | | - | | Cisplatin | | MTT | - | | CD44+ and CD44– cells were resistant to cisplatin, as compared to differentiated spheroids. | | - | | - | |
| Kaseb,  2016  [26] | | CSC-enriched spheroids | | 5 | | 7-11 | | 80 – 100% | | RT | | Colony forming assay,  spheroid migration assay | | - | SF 2.5 Gy: 80%  SF 5 Gy: 75%  Spheroid migration 0 Gy: 2000%;  2.5 Gy: 1400%; 5 Gy: 1100% | | - | | - | |
| Kijima,  2018  [48] | | Organoids | | 5 | | 14 | | 80% | | 5-FU,  Chloroquine | | WST-1 metabolism,  IHC,  FACS | Organoids recapitulated histopathology of the original tumor.  5-FU resistance has increased in secondary (p1) organoids compared to primary (p0) organoids | | IC50 5-FU p0:  1.360 – 0.925 – 0.389 µM  IC50 5-FU p1:  23.60 – 53.62 µM | | - | | - | |
| Tanaka,  2018  [30] | | Organoids | | 43 | | 8 - 30 | | 30.2% | | Cisplatin,  Docetaxel | | Relative organoid area day 1 *vs*. day 8 | Histological patterns, vimentin expression and CD44/ALDH1A1 ratios were similar between organoids and the original tumor. | | Cisplatin IC50: 0.92 – 1.02 µM  Docetaxel IC50: 1.46 – 3.75 nM | | - | | - | |
| Driehuis,  2019  [31] | | Organoids | | 34 | | 42 | | 60% | | Cisplatin,  Carboplatin,  RT | | CellTiter-Glo 3-D Assay | Tumor-specific histopathologic changes were retained in culture.  Organoids contain only transformed tumor cells. | | IC50 cisplatin: 0.5 – 12.8 μM  IC50 carboplatin: 3.0 – 81.9 μM  AUC RT: 238 - 698 | | RT | | 6/7 matched response:  3 positive outcomes with sensitive organoid, 3 no response with non-sensitive organoid. | |
| Robbins, 1991  [77] | | Histocultures  (1-2 mm^3^) | | 15 | | 3-11 | | 67% | | Cisplatin,  5-FU | | ^3^H-TdR | - | | Cisplatin sensitive: 5/10  5-FU sensitive: 4/9  Cisplatin + 5-FU sensitive: 7/8 | | - | | - | |
| Au,  1993  [52] | | Histocultures  (HDRA) | | 83 | | 9 | | 59% | | Cisplatin,  5-FU,  MMC | | ^3^H-TdR | Most histocultures contained areas of viable and necrotic tissue.  Histology of viable regions of the histocultures was similar to that of the fresh tumor. | | Primary tumors mean IC50:  5-FU: 0.68 ± 0.74 µg/ml  Cisplatin: 3.77 ± 2.42 µg/ml  MMC: 0.25 ± 0.13 µg/ml 9/47 tumors not sensitive | | - | | - | |
| Robbins, 1994  [32] | | Histocultures  (HDRA) | | 26 | | 3-11 | | 88% | | Cisplatin | | ^3^H-TdR | - | | 84% reduction in the number of cells incorporating ^3^H-TdR in in drug-treated samples compared to control samples is used as the cutt-off for sensitivity *in-vitro*. | | Cisplatin | | Sensitivity: 71%  Specificity: 78%  PPV: 83%  NPV: 64% | |
| Robbins, 1996  [53] | | Histocultures | | 43 | | 6-9 | | 91% | | Cisplatin | | ^3^H-TdR | - | | Sensitivity overall:  1.5µg/ml: 22%  15 µg/ml: 62%  37.5 µg/ml: 83%  Factor growth inhibition:  Untreated lesions: x 2.44  Recurrent tumors: x 5.56 | | - | | - | |
| Kuh,  1999  [105] | | Histocultures  (1 mm^3^) | | 3 | | 2-4 | | - | | Paclitaxel | | [3H]paclitaxel uptake and efflux | - | | T ½, uptake:  12 nM: 24.2 hours  120 nM: 19.8 hours  1200 nM: 10.4 hours  12,000nM: 5.81 hours  T ½, efflux:  120 nM: 7.45 hours  1200 nM: 3.33 hours | | - | | - | |
| Welters, 1999  [54] | | Histocultures  (3 mm^3^) | | 8 | | 1 | | - | | Cisplatin | | 32-P labeling | - | | Because most of the biopsies were too small to perform analyses at several time points, no adduct levels over time could be measured. | | Cisplatin | | DNA adduct levels partial responder *vs* non- responder: Pt-GG: 27.4 *vs*. 5.1 Pt-AG: 13.2 *vs.* 2.4 | |
| Singh,  2002  [55] | | Histocultures  (HDRA) | | 41 | | 2 | | 98% | | Cisplatin,  5-FU | | MTT | - | | Number of resistant tumors:  13/41 resistant to 5-FU  13/41 resistant to cisplatin  11/41 resistant to both | | Cisplatin,  5-FU,  RT | | 2-year CSS sensitive *vs* not-sensitive:  5-FU: 85% *vs.* 64%  Cisplatin: 86% *vs.* 63%  5-FU + cisplatin: 85% *vs.* 63% | |
| Ariyoshi, 2003  [56] | | Histocultures  (HDRA) | | 19 | | 7 | | 100% | | Cisplatin,  Docetaxel,  5-FU,  BLM  THP,  TXT  ADM | | MTT | - | | Sensitivity rate per drug:  Cisplatin: 78.9%  5-FU: 38.4%  BLM: 21.4%  THP: 7.7%  TXT: 100%  ADM: 0% | | Cisplatin, 5-FU, THP,  BLM | | Accuracy: 78.9%  Sensitivity: 86.7%  Specificity: 50%  TPR: 86.7%  TNR: 50% | |
| Gan,  2006  [106] | | Histocultures  (1 mm^3^) | | 22 | | 3-4 | | 100% | | Paclitaxel | | BrdU labeling,  3-TH, TUNEL, DNA fragmen-tation analysis, IHC | - | | Max. antiproliferation effect: 40%, Max. apoptotic index: 12%  Max. overall effect (max. inhibition of DNA labeling index in non-apoptotic cells): 60% | | - | |  | |
| Hasegawa,  2006  [57] | | Histocultures  (HDRA) | | 49 | | 7 | | 100% | | Cisplatin,  5-FU | | MTT | - | | Cisplatin efficacy rate: 36.7-71.4%  5-FU 120μg/ml *vs*.  300μg/ml efficacy rate:  23.1-57.7% *vs*. 70.8-75.0% | | Cisplatin,  5-FU | | Prediction rate: 77.8%  Sensitivity: 90.9%  Specificity: 57.1%  TPR: 76.9%  TNR: 80.0% | |
| Hasegawa, 2008  [58] | | Histocultures | | 44 | | 7 | | 82% | | Cisplatin,  5-FU | | MTT | - | | Mean I.I. 5-FU: 36.76%  Mean I.I. cisplatin: 35.65%  5-FU sensitivity: 21/44 (58.3%)  Cisplatin sensitivity: 21/44 (58.3%) | | - | | - | |
| Pathak,  2008  [59] | | Histocultures  (HDRA) | | 57 | | 8 | | 91% | | Cisplatin,  5-FU,  MTX | | MTT | - | | cisplatin sensitivity: 52%  5-FU sensitivity: 46%  MTX sensitivity: 52%  Sensitive to one drug: 88% | | Cisplatin,  5-FU,  MTX,  Paclitaxel,  Ifosfamide | | Accuracy: 74%  Sensitivity: 79%  Specificity: 71%  PPV: 69%  NPV: 80% | |
| Gerlach, 2013  [34] | | Histocultures  (350 µm) | | 12 | | 3-6 | | - | | Cisplatin, docetaxel | | IHC | Cultures maintained morphological features and γH2AX expression for up to 6 days compared to original histopathology. | | Control *vs*. cisplatin *vs* docetaxel:  # nuclei: ±400 *vs*. ±125 *vs*. ±150  % caspase-3 positive cells:  ±2% *vs*. ±6% *vs*. ±22% | | - | | - | |
| Yu,  2014  [107] | | Histocultures | | 2 | | - | | - | | 5-ALA-PDT | | FACS | - | | ALDH1 activity:  control: 100%  ALA-PDT treated cell line *vs* tissue slice:  60% *vs*. 45% | | - | | - | |
| Suzuki,  2015  [60] | | Histocultures  (HDRA) | | 28 | | 7 | | 100% | | Cisplatin | | MTT | - | | SUV_max_: 14.04 ± 7.52  I.I.: 50.98 ± 26.6  SUV_max_ was significantly correlated with the I.I. cisplatin (*p*<0.04, R^2^=0.17) | | Cisplatin,  5-FU,  RT | | SUV_max_ ≥10.5 and I.I. cisplatin <50 were significantly correlated with shorter OS | |
| Engelmann,  2020  [61] | | Histocultures | | 13 | | 7-21 | | 100% | | RT | | IHC | Comparable histological and morphological characteristics were observed between primary non-HPV driven tumors and histocultures after 14 days.  Cultures display heterogeneous growth patterns on dermal equivalent | | Irradiation of tissues resulted in a slight increase or decrease in Ki-67 expression compared to control:  Overall: + 0.22%  Non-HPV driven: -5.28%  HPV-driven: +3.89%  2/5 tumors showed increase in apoptotic cells after fractionated irradiation | | RT | | One patient developed local relapse, with the corresponding histoculture showing an invasive growth pattern. | |
| Hattersley,  2012  [41] | | Microdevice | | 23 | | 8 | | 91% | | Cisplatin,  5-FU | | LDH and cytochrome c release, WST-1 metabolism | The nuclei of the tissue 72h after culture appear intact and loss of cell cohesion is minimal.  There was no necrosis in the center of the biopsy. | | % viable cells after treatment:  Control: 72% ± 15.6  5-FU: 45% ± 22.3  Cisplatin: 44% ± 20.2  5-FU + Cisplatin: 30% ± 23.7  All treatments showed a higher release of cytochrome-c than the control samples. (p < 0.01) | | - | | - | |
| Sylvester, 2013  [108] | | Microdevice | | 3 | | 10 | | - | | Cisplatin,  5-FU,  Docetaxel | | LDH release assay, WST-1 metabolism | The combination of LDH release and WST-1 metabolism demonstrated tissue viability within the device. | | Cisplatin alone and combination treatments were significantly different from the untreated controls and 5-FU samples. | | - | | - | |
| Carr,  2014  [62] | | Microdevice | | 35 | | 2-3 | | - | | RT | | LDH and cytochrome c release,  IHC | There was no significant difference between the apoptotic index (AI) of the uncultured and cultured control tissue (p = 0.29). | | AI 0 Gy: ±1%  AI 5 Gy: ±7%  AI 10 Gy: ±15%  AI 20 Gy: ±20%  AI 40 Gy: ±45% (p=0.006) | | - | | - | |
| Hsieh,  2015  [78] | | Microdevice | | - | | 2 | | - | | Cisplatin | | Cell Counting Kit 8, LIVE/DEAD kit, | The metabolic activitity of cells in the 3D culture were higher than in the spheroid culture.  The proliferation of the primary cells in the 3D culture was about 11–101% higher than that in the 2D and spheroid cultures. | | Spheroid cultures might overestimate chemoresistance to cisplatin, the 2D and 3D culture models might overestimate the chemosensitivity to cisplatin  The chemosensitivity of primary cells in the tumor tissue and the 3D culture models showed no statistical difference, indicating that chemosensitivity of the 3D culture was closer to that of the native tumor. | | - | | - | |
| Cheah,  2017  [63] | | Microdevice | | 5 | | 2 | | 100% | | RT,  Cisplatin | | LDH release, IHC, TUNEL | - | | γH2AX: 1/5 sign. response  CK18-LI: 2/5 sign. response  TUNEL: 3/4 sign. response  Ki-67: 0/5 sign. response | | RT,  CRT | | Matched responses for 2/2 patients (for 2/4 markers) | |
| Kennedy, 2019  [64] | | Microdevice | | 18 | | 3 | | 67% | | RT,  Cisplatin | | IHC | The average Ki-67 index decreased in the control sample (7.9% ± 3.5) relative to the pre-culture sample.  No difference in γH2AX and apoptosis between pre-culture and control samples. | | Control *vs*. RT:  BrdU: 13.3% *vs* 7.0%,  Ki-67: 15.3% *vs* 4.0%,  γH2AX: 76.6% *vs*. ± 90%,  Caspase cleaved cytokeratin 18:  ± 3% *vs*. ± 12%  Addition of cisplatin: 1.9-fold increase in apoptotic index | | - | | - | |

5-FU = 5-fluoroucil, RT = Radiotherapy, FACS = Fluorescence-activated Cell Sorting, ALD = Aldehyde Dehydrogenase, CSC = Cancer Stem Cell, MTT = 3-(4,5-Dimethylthiazol-2-yl)-2,5-Diphenyltetrazolium Bromide, SF = Surviving fraction, Gy = Gray, WST-1 = 4-[3-(4-Iodophenyl)-2-(4-nitro-phenyl)-2H-5-tetrazolio]-1,3-benzene sulfonate, IHC = immunohistochemistry, IC50 = half maximal inhibitory concentration, ALDH1A1 = Aldehyde dehydrogenase 1 family, member A1, , AUC = Area under the curve, MMC = Mitomycin C, 3-TH = [^3^H]Thymidine, HDRA = Histoculture Drug Response Assay, PPV = Positive Predictive Value, NPV = Negative Predictive Value, T ½ = Time for the tumor concentration to reach 50% of steady-state level, pt-DNA = Platinum-DNA, CSS = Cause Specific Survival, BLM = Bleomcyin THP: 4-0-tetrahydropyranyl adriamycin, ADM = Adriamycin, TPR = True Positive Ratio, TNR = True Negative Ratio, BrdU = Bromodeoxyuridine, TUNEL = Terminal deoxynucleotidyl transferase dUTP nick end labeling, I.I = Inhibition Index, MTX = Methotrexate, 5-ALA-PDT = 5-aminolevulinic acid photodynamic therapy, SUV = Standardized Uptake Value, OS = Overall survival, LDH = Lactate dehydrogenase, AI = Apoptotic index, CRT = Chemoradiotherapy
