# Supplementary material for: Ex Vivo Culture Models to Indicate Therapy Response in Head and Neck Squamous Cell Carcinoma
Source: Cells. 2020 Nov 23;9(11):2527. doi: 10.3390/cells9112527 (PMC7700693; doi:10.3390/cells9112527)
Supplement: Supplementary file 1 [file cells-09-02527-s001.zip › Table S2.docx]

**Table S2.** Overview of included studies describing ex-vivo culture models of HNSCC for immunotherapy and targeted therapy sensitivity testing.

| Authors,  year | Culture technique | Patients  (N) | Culture duration  (days) | Culture  success  (%) | Ex-vivo treatment | Response read-out method | Preservation of tissue parameters in culture | Main results of treatment |
| --- | --- | --- | --- | --- | --- | --- | --- | --- |
| Heimdal,  2001  [20] | Multicellular Spheroids | 3 | 10-14 | - | Anti-CD14 mAb | ELISA | - | Addition of anti-CD14 mAb reduces LPS-induced IL-6 secretion. The addition of anti-CD14 mAb to co-cultures of monocytes and spheroids increased the IL-6 response as tested in co-cultures from three donors. |
| Heimdal,  2001  [72] | Multicellular Spheroids | 11 | 10-14 | - | anti-MCP-1 mAb | ELISA | - | Addition of anti-MCP-1 mAb decreases co-culture-induced monocyte IL-6 secretion in 4/5 cultures. compared to control cultures. |
| Olsnes,  2002  [73] | Multicellular Spheroids | 10 | 10-14 | 100% | PFA,  D-galactose,  D-glucose,  D-mannose,  D-fructose,  anti-CD29 mAb | ELISA | - | Monocyte IL-6 secretion was inhibited by PFA, D-Glucose, D-mannose and anti-CD29 treatment. |
| Kross,  2007  [65] | Multicellular Spheroids | 18 | 14 | > 90% | LLME | ELISA,  LIVE/DEAD kit,  BrdU labeling | Nearly 100% of the spheroid surface consisted of live cells, indicating viability after 14 days of culture in vitro. | Mean IL-6 production in 168 h, control *vs* treated:  ± 17.500 *vs*. ± 5000 pg/mL  Mean MCP-1 production in 168 h, control *vs* treated: ± 7000 *vs*. ± 1000 pg/mL |
| Baumeister,  2009  [109] | Multicellular Spheroids | 30 | 21 | 50-100% | Benzopyrene diolepoxide (BPDE),  TGF-a | Comet-  assay | Overall cell viability was >90%. | Non-treated OTM: 0.9  Control *vs*. tumor BPDE: 7.5 *vs*. 9.4  Control *vs*. tumor BPDE + TGF-a: 7.0 *vs*. 5.7  Control *vs*. tumor hydrogen: 9.7 *vs*. 12.1  Control *vs*. tumor hydrogen + TGF-a:  8.8 *vs*. 11.4  Control *vs* tumor DNA repair capacity:  57.5% *vs*. 45.0% |
| Leong,  2014  [51] | Multicellular Spheroids | 3 | 4-9 | - | Gefitinib  NVP-AEW541 | FACS | - | Tumor spheres were more resistant to all treatment regimens than cells grown in monolayer culture.  Although the ALD+ fractions were significantly reduced after treatment with the individual inhibitors, maximal reduction was seen after  combined treatment with both drugs |
| Kloss,  2015  [66] | Multicellular Spheroids | 5 | 11-14 | 100% | Cetuximab | Cytometric Bead Array,  fluorescent  microscopy,  FACS | - | When cetuximab was absent the NK cells showed  clearly impaired and disordered “effector- to  target” interactions and decreased both cancer  cell cluster infiltrations and cancer cell killing. |
| Sun,  2014  [67] | CSC-enriched spheroids | 3 | 6 | - | c-Met inhibitor (PF-2341066) | Sphere forming ability | Immunofluorescent staining showed that the spheres have high expression levels of several known CSC markers | Sphere formation was inhibited in a dose-dependent manner.  CSC cells were more sensitive to PF-2341066 than to docetaxel. In contrast, differentiated cells show the opposite effect. |
| Driehuis,  2019  [31] | Organoids | 34 | 42 | 60% | Nutlin-3  Cetuximab,  Alpelisib,  Vemurafenib  Everolimus,  AZD4547 Niraparib | CellTiter-Glo 3-D Assay | Tumor-specific histopathologic changes from the primary material were retained in culture.  The organoids contain only epithelial tumor cells, not the immune, connective tissue, or vessel elements. | IC50 Nutlin-3: 0.5 – 22.6 µM  AUC cetuximab: 93.94 - 180.7  IC50 alpelisib: 0.12 – 4.12 µM  IC50 everolimus: 0.00 – 19.83 µM  IC50 AZD4547: 0.67 – 28.38 µM  IC50 Niraparib: 4.24 – 25.66 µM |
| Driehuis,  2019  [76] | Organoids | 7 | 7-14 | - | PDT  (7D12-PS,  7D12-9G8-PS, Cetuximab-PS) | CellTiter-Glo 3-D Assay | EGFR expression levels in organoids were comparable to EGFR levels of primary patient material samples. | AUC of 7D12-PS: 11,490 – 17,657  AUC of 7D12-9G8-PS: 12,167 – 53,548  AUC of Cetuximab-PS: 36,296 – 66,487  EGFR expression levels correlate with response to EGFR-targeted PDT:  R^2^ = 0.44 (7D12)  R^2^ = 0.27 (7D12-9G8)  R^2^ = 0.48 (Cetuximab) |
| Dean,  2010  [68] | Histocultures  (800-1000 µm) | 22 | 3 | 86,4% | Anti-EMMPRIN mAb,  Cetuximab | ATP viability assay,  TUNEL | Cultures had excellent viability over 72h.  Less than 5% of any specimen showed necrosis. | Average ATP level anti-EMMPRIN *vs*. Cetuximab:  57% *vs*. 45% (Control: 100%) (p = 0.13)  Apoptosis was increased in CNTO treated cultures (77%) *vs*. controls (30%). |
| Gerlach,  2013  [34] | Histocultures  (350 µm) | 12 | 3-6 | - | Cetuximab | LDH release,  IHC,  TUNEL | Slice cultures maintained morphological features for up to 6 days as compared to the original diagnostic histopathology.  No change of γH2AX positivity was visible at any of the tested time points. | Number of nuclei control *vs*. Cetuximab:  ± 400 *vs*. ± 25  Percentage of caspase-3 positive cells control *vs*. Cetuximab: ± 2% *vs*. ± 5% |
| Radhakrishnan, 2013  [110] | Histocultures | 22 | 3 | - | Rapamycin  MK-2206 | Cell Counting Kit 8,  WST assay,  IHC | - | Cell viability Rapamycin *vs*. MK-2206 *vs*. combination (control = 100%):  120% *vs*. 100% *vs*. 85%  Apoptotic cells Rapamycin *vs*. MK-2206 *vs*. combination (control = 100%):  15% *vs*. 14% *vs*. 19% |
| Saussez,  2014  [111] | Histocultures | 5 | 1 | 60% | Glutamate,  Dopamine,  GnRH-II | FACS | The T cells of three patients varied in their basal level of spontaneous and chemotactic migration. | All neurotransmitters increased T-cell migration, CD3zeta expression, and CD3 epsilon expression |
| Dayekh,  2014  [112] | Histocultures  (1 mm) | 4 | 2 | 100% | Monensin | qRT-PCR, | - | 3/4 cultures showed >2-fold increase of HMG-CoA reductase mRNA levels after Monensin treatment.  2/4 cultures showed > 4-fold increase of ATF3 mRNA levels after Monensin treatment. |
| Freudlsperger, 2014  [35] | Histocultures  (300-350 µm) | 15 | 6 | - | LY294002 | IHC | Histological staining confirmed preservation of tissue architecture.  The cultures showed almost 100% Ki-67 staining and few apoptotic cells. | Expression after treatment with LY294002 *vs*. RT *vs*. LY294002 + RT (control 100%):  p-AKT:± 65% *vs*. ± 135% *vs*. ± 55%  p-H2AX: ± 80% *vs*. ± 900% *vs*. ±1700%  Ki-67: ± 80% *vs*. ± 70% *vs*. ±35% |
| Bourouba,  2015  [113] | Histocultures | 20 | 1 | - | Anti-TNFa mAb,  NOS2 inhibitor | IHC,  NO2 measurement (Griess reaction) | - | Average NO2 production anti-TNFa *vs*. 1400W (control = 100%): ± 65% *vs*. ± 55%  Ki-67 % proliferation index untreated *vs*. anti-TNFa: ± 45% *vs*. ± 33% (p < 0.05) |
| Peria,  2015  [36] | Histocultures  (300 μm) | 5 | 3 | 80% | Cetuximab  Sorafenib | IHC | After 72 hours, an increase in tumor necrosis was observed in cultured tumor slices. After 48h, proliferation decreased by 30-70%. | Average % Ki-67 positive cells, control *vs*. Cetuximab *vs*. Sorafenib:  ± 25% *vs*. ± 15% *vs*. ± 21% |
| Rauth,  2016  [69] | Histocultures  (2-3 mm^3^) | 5 | 3 | 100% | Lupeol | IHC | Key components of tumor microenvironment were found to be intact up to 3 days | Tumor cell content control *vs* Lupeol:  ±70% *vs*. ±45% (p <0.05)  Ki-67 positive cells control *vs* Lupeol:  ± 15% *vs*. ± 2% (p < 0.01) |
| Affolter,  2016  [70] | Histocultures  (800-1000 μm) | 9 | 6 | 100% | MEK inhibitor  PD-0325901, | IHC | The number of Ki‐67 positive tumor cells was 5% to 7.5% in nontreated cultures. In 1 culture, 75% of all cells were positive for Ki‐67 in the control.  γH2AX expression levels varied widely between 10% and 95% | Expression after treatment with 0 μM PD-0325901 + 5 Gy *vs*. 20 μM PD-0325901+ 5 Gy:  pERK: 27.8% *vs*. 4.4%  Ki-67: 8.1% *vs*. 1.8%  γH2AX: 43.1% *vs*. 43.1% |
| Donnadieu,  2016  [71] | Histocultures  (300 μm) | 18 | 2 | 78% | 8 different drugs, see “result treatment column” | IHC | - | Average % of cell inhibition (control = 100%):  Rapamycin: 77.1% Sorafenib: 65.7%  Cetuximab: 73.4% Erlotinib: 75.9%  Masatinib: 70.5% Ponatinib: 74.2%  Afatinib: 60.9% Tivantinib: 80.9% |
| Bhattacharyya, 2017  [114] | Histocultures | 20 | 3 | - | Lupeol | IHC | - | % proliferation control *vs*. Lupeol: 100% *vs*. ± 55%  % apoptotic cells control *vs*. Lupeol: ± 1% *vs*. ± 11% |
| Baird,  2018  [115] | Histocultures  (2mm^3^) | 5 | 1 | 100% | STING ligands | Cytokine secretion analysis,  IHC | - | Treatment with STING ligands resulted in increases of IFNα secretion from the explant  4/5 patients showed increased in secretion of CCL3 after treatment with STING ligands. |
| Carter,  2019  [116] | Histocultures  (1-2 mm^3^) | 10 | 2 | 100% | BCL-2 family inhibitors (A-1331852 and S63845) | IHC | - | % of cleaved PARP positive tumor cells:  Control: 8%  A-1331852: 7%  S63845: 6%  A-131852 + S63845: 36% |
| Al-Samadi,  2019  [42] | Microdevice | 5 | 3 | - | IDO 1 inhibitor, PD-L1 antibody | Fluorescent microscopy-based cell counting | - | AUC # of infiltrated immune cells  Control *vs*. IDO 1 *vs*. PD-L1:  Patient 4: ± 550 *vs*. ± 850 *vs*. ± 400  Patient 5: ± 0 *vs*. ± 250 *vs*. ± 0  AUC cancer cell proliferation rate:  Patient 4: ± 1.0 *vs*. ± 0.85 *vs*. ± 0.4  Patient 5: ± 1.0 *vs*. ± 0.7 *vs*. ± 0.8 |

mAb = Monoclonal antibody, ELISA = Enzyme-Linked Immunosorbent Assay, LPS = Lipopolysacharide , IL-6 = Interleukin-6, MCP-1 = Monocyte Chemoattractant Protein-1, PFA = Paraformaldehyde, LLME = L-leucine-methylester, BrdU = Bromodeoxyuridine, TGFa = Transforming growth factor-alpha, OTM = Olive Tail moment, FACS = Fluorescence-activated Cell Sorting, ALD = Aldehyde dehydrogenase, NK cells = Natural Killer cells, CSC = Cancer Stem Cell, IC50 =. , PDT = Photodynamic therapy, PS = Photosensitizer, EGFR = Epidermal growth factor receptor, AUC = Area Under the Curve, EMMPRIN = extracellular matrix metalloproteinase inducer, ATP = Adenosine triphosphate, LDH = Lactate dehydrogenase, TUNEL = Terminal deoxynucleotidyl transferase dUTP nick end labeling, GnRH-II = gonadotropin-releasing hormone-II, RT-PCR Real time polymerase chain reaction ,ATF3 = ctivation of transcription factor 3, IHC = immunohistochemistry, RT = Radiotherapy, Gy = Gray, TNFa = Tumor necrosis factor-a, NOS2 = Nitric oxide synthase 2, STING = Stimulator of interferon genes, IFNa = Interferon-a, CCL3 = Chemokine (C-C motif) ligand 3, PARP = Poly (ADP-ribose) polymerase, IDO1 = Indoleamine 2,3-dioxygenase, PD-L1 = Programmed death-ligand 1
